# RSK1 activation promotes invasion in nodular melanoma

**DOI:** 10.1186/1479-5876-13-S1-O2

**Published:** 2015-01-15

**Authors:** Amel Salhi, Joshua A Farhadian, Keith M Giles, Eleazar Vega-Saenz de Miera, Ines P Silva, Caitlin Bourque, Karen Yeh, Sagar Chhangawala, Jinhua Wang, Fei Ye, David Y Zhang, Eva Hernando, Yariv Houvras, Iman Osman

**Affiliations:** 1 The Ronald O. Perelman Department of Dermatology, New York University School of Medicine, New York, NY, USA; 2 Department of Pathology, New York University School of Medicine, New York, NY, USA; 3 Interdisciplinary Melanoma Cooperative Group, New York University School of Medicine, New York, NY, USA; 4 Departments of Surgery and Medicine, Weill Cornell Medical College, New York, NY, USA; 5 NYULMC Perlmutter Cancer Center, NYU Center for Health Informatics and Bioinformatics, New York, NY, USA; 6 Department of Pathology, Mount Sinai School of Medicine, New York, NY, USA

## Background

The two major melanoma histologic subtypes, superficial spreading and nodular melanomas, are believed to differ in their speed of dermal invasion but to converge biologically once they invade and metastasize. Here, we tested the hypothesis that distinct molecular alterations arising in primary melanoma cells might persist as these tumors progress to invasion and metastasis.

## Materials and methods

Expression of 141 signaling proteins was evaluated by protein pathway array in 3 Radial Growth Phase (RGP)/SSM and 3 Vertical Growth Phase (VGP)/NM cell lines. The impact of p90-ribosomal-S6-kinase (RSK1) and its inhibition on proliferation, migration and invasion was assessed in SSM and NM cell lines, and confirmed using NM cells treated with a RSK inhibitor (BI-D1870) in microarray profiling studies. The effect of constitutive RSK1 activation in vivo was further studied using a zebrafish model.

## Results

We show that p90-ribosomal-S6-kinase (RSK1) was significantly hyper-activated in human melanoma lines and metastatic tissues derived from nodular compared with superficial spreading melanoma. RSK1 was constitutively phosphorylated at Ser-380 in nodular but not superficial spreading melanoma and was not directly correlated with BRAF or MEK activation. Nodular melanoma cells were more sensitive to RSK1 inhibition using both siRNA and pharmacological inhibitor BI-D1870 compared with superficial spreading cells. In addition, gene expression microarray analyses revealed that RSK1 orchestrates a program of gene expression that promotes cell motility and invasion. Our data also demonstrate a differential overexpression of the pro- metastatic MMP-8 and TIMP-1 in metastatic nodular compared to metastatic superficial spreading melanoma. Finally, using an in vivo zebrafish model, constitutive RSK1 activation increased melanoma invasion.

## Conclusions

Together, our data reveal a novel role for activated RSK1 in the progression of nodular melanoma, and suggest that melanoma originating from different histological subtypes may be biologically distinct and that these differences are maintained as the tumors invade and metastasize.

